# Fertility preservation counselling in women with cancer: knowledge, preferences, and unmet needs from a cross-sectional survey

**DOI:** 10.3389/fendo.2026.1926475

**Published:** 2026-10-06

**Authors:** Giacomo Corrado, Drieda Zace, Inge Peters, Paola Villa, Luigi Petrella, Alessia Barreca, Lucrezia Massaro, Letizia Lafuenti, Erica Silvestris, Stefano Canosa, Cristina Fabiani, Daniela Rosaria Pia Chieffo, Anna Fagotti, Maria Luisa Di Pietro

**Affiliations:** 1 Gynecologic Oncology Unit, Department of Woman’s and Child Health, Fondazione Policlinico Universitario Agostino Gemelli Istituto di Ricovero e Cura a Carattere Scientifico (IRCCS), Rome, Italy; 2 Infectious Disease Clinic, Department of Systems Medicine, University of Rome Tor Vergata, Rome, Italy; 3 Centre for Research and Studies on Procreative Health, University Department of Life Sciences and Public Health, Università Cattolica del Sacro Cuore, Rome, Italy; 4 Department of Gynecology, Antoni van Leeuwenhoek-Netherlands Cancer Institute, Amsterdam, Netherlands; 5 Local Health Authority ASST Nord Milano, S. C. Hygienic Sanitary Processes of The Territorial Center Asst Nord Milan, Milan, Italy; 6 Department of Women, Mother and Neonate, “Vittore Buzzi” Children’s Hospital, University of Milan, Milan, Italy; 7 Clinical Psychology Unit, Fondazione Policlinico Universitario A. Gemelli Istituto di Ricovero e Cura a Carattere Scientifico (IRCCS), Rome, Italy; 8 Gynecologic Oncology Unit, Istituto di Ricovero e Cura a Carattere Scientifico (IRCCS) Istituto Tumori ‘Giovanni Paolo II’, Bari, Italy; 9 Obstetrics and Gynecology 1U, Physiopathology of Reproduction and IVF Unit, Department of Surgical Sciences, Sant Anna Hospital, University of Turin, Turin, Italy; 10 Regional Oncofertility Center, Sandro Pertini Hospital, Rome, Italy; 11 Department of Life Sciences and Public Health, Section of Gynecology and Obstetrics, Università Cattolica del Sacro Cuore, Rome, Italy; 12 Department of Life Sciences and Public Health Section of Hygiene, Fondazione Policlinico Universitario A. Gemelli Istituto di Ricovero e Cura a Carattere Scientifico (IRCCS), Rome, Italy

**Keywords:** cancer survivorship, counselling, fertility preservation, oncofertility, reproductive intention

## Abstract

**Background:**

Fertility preservation (FP) is a critical survivorship issue for young women with cancer, yet gaps persist between guidelines and clinical practice. This study assesses knowledge, attitudes, and unmet needs among women referred for specialist counseling.

**Methods:**

An observational cross-sectional survey was conducted (November 2022 – June 2026) among women referred to a single tertiary oncofertility center in Rome. A Delphi-informed online questionnaire was completed before the clinical visit. Descriptive statistics and exploratory multivariable logistic regression examined post-diagnosis intention.

**Results:**

Among 284 women invited to participate, 260 completed the survey (response rate, 91.5%); 240 patients with a confirmed cancer diagnosis were included in the final analysis (median age, 32 years). Overall, 69.6% were interested in FP, and 94.2% recognized that gonadotoxic treatments could impair fertility. While 89.6% had heard of FP, only half received this information from healthcare professionals, and 24.2% knew of no specific options. Over half reported concerns regarding treatment delay, safety, effectiveness, and cost. Before diagnosis, 73.7% planned to have children; post-diagnosis, positive intention remained common, while those previously without intentions shifted toward uncertainty. In the 188-case model, pre-diagnosis intention showed the strongest association (OR 11.64, 95% CI 4.45–30.48). Given limited model performance (pseudo-R²=0.19), estimates warrant caution.

**Conclusions:**

In this single-center referred cohort, informational gaps and uncertainty remained despite high awareness. Findings confirm and contextualize prior evidence in an Italian tertiary setting and may inform similar referral pathways, but require validation in broader populations.

## 1 Introduction

Cancer in children, adolescents, and young adults is a major global health challenge due to its impact on survival and future reproductive potential (1, 2). According to GLOBOCAN 2022 (Global Cancer statistics which functions as an online database created by the International Agency for Research on Cancer - IARC), 928,991 new cancer cases were diagnosed worldwide in females aged 0–39, led by breast (26.2%), thyroid (18.6%), cervical (11.3%), leukemia (5.8%), and ovarian cancer (4.3%). This group saw 240,642 deaths, with breast cancer being the leading cause (20.2%) (3). Improved survival has shifted focus toward long-term quality of life and reproductive potential (4–6). Fertility is now a primary survivorship issue, especially as the age at first birth rises to 32–33 years in countries like Italy, Spain, and Korea (7). Consequently, more women face cancer therapies before completing their reproductive plans, risking premature ovarian insufficiency and permanent infertility.

Gonadotoxicity depends on patient age, baseline ovarian reserve, and treatment type (8). Alkylating agents and stem cell transplant conditioning carry the highest risk, while antimetabolites are generally less damaging (8, 9). Pelvic irradiation is highly detrimental: oocyte damage occurs even at low doses and irreversible ovarian failure is age-dependent (10); uterine damage may also occur, particularly after prepubertal exposure, potentially impairing uterine growth, vascularity and future obstetric outcomes (11). In gynecological cancers, surgery and radiotherapy may directly compromise fertility.

Recovery of ovarian function is variable and influenced by age and treatment intensity (9, 12). Crucially, the resumption of menses does not guarantee preserved fertility (13). Biomarkers like anti-Müllerian hormone may show limited recovery (14). In breast cancer, menstrual function recovers in up to 69% of cases (15), particularly in younger women within two years, but is less likely after stem after stem cell transplantation for hematological malignancies (16, 17).

The field of fertility preservation (FP) has evolved significantly over twenty years (18, 19). Established strategies include embryo, mature oocyte, and ovarian tissue cryopreservation, as well as ovarian transposition and conservative surgery (9, 20, 21). Despite ASCO (American Society of Clinical Oncology) and ASRM (American Society for Reproductive Medicine) recommendations, gaps remain between guidelines and practice. Current guidance (9) mandates discussing infertility risks and offering timely FP referrals before treatment. However, access remains uneven and referral is often delayed, and patients may receive incomplete information while coping with a recent diagnosis (9). Reported barriers include limited knowledge, fear of treatment delay, financial concerns and limited provider expertise (22–28). Within shared decision-making, reproductive preferences interact with available information, perceived feasibility, practical barriers and psychosocial stress (29, 30). Pre-diagnosis intentions provide the preference context; this study aimed to confirm and contextualize these factors in an Italian tertiary setting and explore their associations.

## 2 Methods

### 2.1 Study design and setting

This observational cross-sectional survey was conducted among women referred for fertility preservation with a confirmed cancer diagnosis between November 2022 and June 2026 at the Oncological Gynecology Unit (Oncofertility Centre) of the Fondazione Policlinico Universitario A. Gemelli IRCCS, Rome, in collaboration with the Centre for Research and Studies on Reproductive Health, Università Cattolica del Sacro Cuore, Rome.

Inclusion criteria were women of reproductive age being diagnosed with any type of cancer who were scheduled for a fertility preservation consultation and provided informed consent. Exclusion criteria included women who had previously undergone a fertility preservation consultation. Participants were recruited consecutively throughout the study period.

Eligible patients received an email with a link to the online questionnaire prior to their fertility preservation consultation and were asked to complete it before the visit. The questionnaire, available in Italian, was administered via a secure online platform, and responses were recorded anonymously. Electronic informed consent was obtained before accessing the questionnaire. This design aimed to minimize potential influence from the consultation itself and to capture women’s baseline views, concerns, and informational needs.

The study was conducted and reported in accordance with the Consensus-Based Checklist for Reporting of Survey Studies (CROSS) (31) and was approved by the Ethics Committee of Lazio 3 (n.4942 of 09/02/2023).

### 2.2 Questionnaire development

The questionnaire was specifically developed for the study using a Delphi procedure. Item generation was informed by a review of the literature on fertility, oncofertility and preconception health. The research string included keywords such as “fertility preservation”; “cryopreservation”; “ovarian tissue cryopreservation”; “oocyte cryopreservation”; “oncofertility”; “fertility concern”; “tumor”; “cancer”; “malignancy”; “chemotherapy”; “decision-making”; “counselling”; “information provision”. On the basis of the needs identified in the systematic review, the questionnaire was structured into five domains covering socio-demographic information, clinical history, fertility and consultation, reproductive attitudes, and personal implications.

The questionnaire was refined through a two-round Delphi process involving 15 multidisciplinary experts. Items were rated for relevance on a 5-point Likert scale (where 1 = ‘strongly disagree, this question should not be included in this questionnaire/it is irrelevant’ and 5 = ‘strongly agree, this question is relevant and should be included in the questionnaire’), and mean score, standard deviation, and content validity index (CVI) were calculated for each item. Items with a CVI >79% were retained, those with a CVI between 70% and 79% were revised, and those with a CVI <70% were considered for removal. In round one, 13 experts participated; items with low relevance were revised or removed on the basis of quantitative ratings and qualitative feedback. In round two, 9 experts reviewed the revised version, and consensus was achieved.

The final questionnaire comprised 50 items across five domains: socio-demographic information (n = 6), clinical history (n = 12), fertility and consultation (n = 20), reproductive attitudes (n = 7), and personal implications (n = 5), using single- and multiple-response items and required 15–20 minutes to complete. The survey assessed FP intentions, awareness of gonadotoxic effects and FP options, counselling timing and sources, FP-related concerns, medical decision-making preferences, and emotional or relational issues (**Supplementary Material**, **Supplementary Table S1**). The instrument was a multidomain survey of factual, categorical and preference items rather than a single latent psychometric scale; the Delphi procedure was used to establish content validity. Its feasibility was assessed during the study by monitoring questionnaire completion and the occurrence of missing or skipped items. Participants were generally able to complete the questionnaire within the study visit, with no particular difficulties or concerns regarding its length or comprehensibility, confirmed also by the high overall questionnaire response rate (91.5%). Completeness of the returned questionnaires was assessed by examining the frequency and distribution of missing or skipped responses across individual items. The overall proportion of missing responses was less than 5%. Formal pilot testing and psychometric validation were not performed. Given its 50-item length shortly after diagnosis, respondent burden is a potential limitation and is considered in the Discussion.

### 2.3 Statistical analysis

Data were summarized using descriptive statistics. Categorical variables were reported as absolute frequencies and percentages, and continuous variables as means with standard deviations, when appropriate. For multiple-response items, each selected option was disaggregated and counted separately, and percentages were calculated on the total number of recorded responses for that item. According to the protocol, inferential analyses were originally planned to explore associations between intention to undergo fertility preservation and selected demographic or clinical variables, using parametric or non-parametric tests as appropriate and chi-square or Fisher’s exact test for categorical comparisons. A multivariable logistic regression model explored associations with post-diagnosis reproductive intention; clinically relevant variables were entered. Complete-case analysis included 188/240 participants; cases with missing data were excluded; analyses used Stata 18.

## 3 Results

Of 284 women invited to participate, 260 provided consent, yielding a response rate of 91.5%. Among the 260 participants, 20 did not have a cancer diagnosis (including hereditary cancer predisposition syndromes and non-oncological conditions) and were excluded, whereas 240 had a cancer diagnosis and were included in the final analysis. The median age at the time of consultation was 32 years (IQR 27-37), and most respondents were Italian (85.8%). Regarding relationship status, 75.0% were in a relationship, 20.8% were single and relationship status was unknown for the remaining patients. Educational attainment was generally high, with 39.2% holding a university degree and 22.9% having completed postgraduate education. More than half of the participants were employed full-time (54.6%) (**Table 1** and **Supplementary Material**, **Supplementary Table S2**).

**Table 1:** Sociodemographic and clinical characteristics of women with an oncological diagnosis referred for fertility preservation counselling VARIABLE N (%).

CONSENT	240/240 (100)
AGE IN YEARS (AT THE TIME OF CONSULTATION)	Median 32 (IQR 27-37)
NATIONALITY	
ITALIAN	206/240 (85.8)
OTHERS	34/240 (14.2)
RELATIONSHIP	
NO, I AM SEPARATED/DIVORCED	1/240 (0.4)
NO, I AM SINGLE	49/240 (20.4)
YES, I AM CASUALLY DATING A PARTNER	10/240 (4.2)
YES, I AM IN A STABLE RELATIONSHIP	61/240 (25.4)
YES, I AM COHABITING/LIVING WITH A PARTNER	69/240 (28.7)
YES, I AM MARRIED	40/240 (16.7)
I PREFER NOT TO ANSWER	10/240 (4.2)
LEVEL OF EDUCATION	
MIDDLE SCHOOL DIPLOMA	15/240 (6.2)
HIGH SCHOOL DIPLOMA	76/240 (31.7)
UNIVERSITY DEGREE	94/240 (39.2)
POSTGRADUATE EDUCATION (E.G. MASTER’S DEGREE, PHD, ETC.)	55/240 (22.9)
EMPLOYMENT STATUS	
NO	68/240 (28.3)
YES, FULL-TIME	131/240 (54.6)
YES, PART-TIME	34/240 (14.2)
I PREFER NOT TO ANSWER	7/240 (2.9)
DISEASE	
BORDERLINE OVARIAN CANCER	34/240 (14.2)
INVASIVE OVARIAN CANCER	37/240 (15.4)
ENDOMETRIAL CANCER	12/240 (5.0)
UTERINE CANCER	2/240 (0.8)
CERVICAL CANCER	12/240 (5.0)
VULVAR CANCER	1/240 (0.4)
BREAST CANCER	86/240 (35.9)
HEMATOLOGICAL MALIGNANCY	35/240 (14.6)
COLORECTAL CANCER	6/240 (2.5)
LUNG CANCER	1/240 (0.4)
BRAIN TUMOR	3/240 (1.3)
SKIN CANCER	2/240 (0.8)
CANCER OF UNCERTAIN ORIGIN	1/240 (0.4)
UNSPECIFIED CANCER	8/240 (3.3)
STAGE OF DISEASE	
I	47/240 (19.6)
II	33/240 (13.7)
III	28/240 (11.7)
IV	7/240 (2.9)
UNKNOWN	125/240 (52.1)
AGE AT DIAGNOSIS IN YEARS *AVAILABLE IN 239/240	Median 32 (IQR 26-37)
FAMILY HISTORY OF CANCER	
YES	142/240 (59.2)
NO	83/240 (34.6)
UNKNOWN	15/240 (6.2)
HEIGHT IN CM	Mean 164.6 (±6.4)
PRE-DIAGNOSIS WEIGHT IN KG *AVAILABLE IN 236/240	Median 60 (IQR 53-65)
CURRENT WEIGHT IN KG *AVAILABLE IN 238/240	Median 60 (IQR 53-65)
BMI CATEGORY *AVAILABLE IN 238/240	
UNDERWEIGHT (<18.5 KG/M2)	28/238 (11.8)
HEALTHY WEIGHT (18.5-24.9 KG/M2)	157/238 (66.0)
OVERWEIGHT (25.0-29.9 KG/M2)	36/238 (15.1)
OBESITY (≥ 30.0 KG/M2)	17/238 (7.1)

### 3.1 Clinical history

Breast cancer was the most frequent diagnosis (35.9%), followed by invasive ovarian cancer (15.4%), hematological malignancies (14.6%), borderline ovarian tumors (14.2%), endometrial cancer (5.0%) and cervical cancer (5.0%). Disease stage was unknown in 52.1% of cases; among known stages, stage I was the most frequent (19.6%). Median age at diagnosis was 32 years (IQR 26-37), and the median time from cancer diagnosis to first fertility preservation consultation was 4 weeks (IQR 2–14). Most patients had never been pregnant (80.8%) and did not have children (86.7%). Most patients had no comorbidities (74.4%) and had a healthy body mass index between 18.5 and 24.9 kg/m² (66.0%) (**Table 1**; **Supplementary Material**, **Supplementary Table S2**). Among those who reported at least one comorbidity, hypothyroidism/hyperthyroidism was the most common condition (9.3%).

### 3.2 Fertility and consultation

Overall, 69.6% of participants stated that they would be interested in starting a fertility preservation procedure at the time of the consultation. This interest appeared to develop in a context of generally high awareness that anticancer treatment may impair fertility: most women believed that chemotherapy and/or radiotherapy could reduce fertility (94.2%). However, perceptions of treatment-related fertility impairment were not uniform. While 39.2% of participants recognized that chemotherapy/radiotherapy may compromise fertility but not invariably, 30.0% believed that fertility is always affected, and 30.4% were uncertain.

Consistently, 89.6% had heard about fertility preservation; among them, 48.4% had heard about it before the cancer diagnosis and 51.6% afterwards. Nevertheless, the pathways through which this information was acquired were variable. Only about half of participants first received information on fertility preservation from healthcare professionals, including oncologists after diagnosis (23.6%) and specialists at the time of diagnosis (22.0%), whereas the remainder relied on non-medical sources such as friends or relatives (21.4%) and media sources (21.2%). Among those who were aware of available strategies, knowledge was largely concentrated on oocyte cryopreservation (64.2%), whereas awareness of alternative options remained very limited, particularly for ovarian tissue cryopreservation (7.1%) and embryo cryopreservation (3.7%). Notably, almost one in four participants (24.2%) reported no knowledge of any fertility preservation option.

This informational gap was reflected in the high value attributed to counselling. Receiving information about fertility preservation was rated as very or extremely important by 80.8% of women. More than half of respondents (55.0%) considered the gynecologist and/or reproductive specialist the most appropriate professional to provide fertility preservation information, whereas 25.4% selected the oncologist. Most women believed that all women diagnosed with cancer should receive information on fertility preservation (57.9%), while 32.1% restricted this to women of reproductive age. Regarding decisional style, half of respondents preferred to make the decision themselves after considering the doctor’s opinion (49.6%), while the other half (46.7%) preferred shared decision-making with the doctor.

Despite this strong interest and the perceived importance of counselling, concerns about fertility preservation remained common (55.4%): the most frequent concerns were delay in the initiation of cancer treatment (29.3%), safety (22.6%), costs (19.5%) and effectiveness (18.8%). In line with this, when asked whether fertility preservation could influence cancer treatment, 34.6% answered yes, 22.1% no, and 43.3% did not know. Among those who answered yes, the perceived mechanisms were mainly delay in the start of cancer treatment (45.8%) and possible complications of ovarian tissue or oocyte retrieval procedures (43.4%) (**Supplementary Material**, **Supplementary Table S2**).

### 3.3 Reproductive attitudes

Before the cancer diagnosis, 73.7% of women with an oncological diagnosis had planned to have children, whereas 26.3% had not. After diagnosis, the proportion with positive reproductive intentions remained similar overall (51.3% in the future and 22.1% as soon as possible), but the group without prior childbearing intentions shifted predominantly toward uncertainty: 23.3% were unsure and only 3.3% reported no future reproductive intention. Only 34.6% considered foster care or adoption. Among women who had a partner, decision-making regarding fertility preservation was often shared, with 57.4% reporting that their partner was very or extremely involved, but notably 13.2% reported no partner involvement. Among women who did not wish to have children after diagnosis, the most common reasons were fear of transmitting cancer to their children because of genetic predisposition or previous chemotherapy exposure (29.6%), concern that pregnancy might increase the risk of recurrence (25.0%), and fear of not being able to care for a child in case of recurrence (20.4%) (**Supplementary Material**, **Supplementary Table S2**).

### 3.4 Personal implications

At the time of the survey, approximately half of all reported thoughts were centered on start of cancer treatment (17.7), future parenthood including fear of not being able to have children (17.1%), fertility preservation options (15.6%) and thoughts reflecting resilience and coping capacity (15.1%). After diagnosis, the factors most frequently perceived as helpful were relational support from friends and family (31.5%) and psychological support (28.5%). In line with this, 79.6% of women considered psychological support important at that time, underscoring the central role of emotional care alongside reproductive counselling.

The impact of cancer diagnosis on couple relationships was not uniform. Half of participants (50.0%) reported no change in their relationship, whereas a positive impact was described twice as often as a negative one (18.4% vs 10.8%). For the fertility preservation consultation, the preferred accompaniment was the partner (38.3%), followed by family members (28.4%) and attending alone (21.7%) (**Supplementary Material**, **Supplementary Table S2**).

### 3.5 Factors associated with post-diagnosis reproductive intention

Among women with an oncological diagnosis, post-diagnosis reproductive intention was significantly associated with age. Mean age differed across intention groups (ANOVA p<0.001), with women wishing to have children as soon as possible being the oldest on average (median 36 years, IQR 33-40), followed by those who were uncertain (median 33 years, IQR 27.5-37.5), those reporting no future desire for pregnancy (median 31 years, IQR 23-39), and those wishing to have children in the future (median 30 years, IQR 26-34) (**Figure 1**).

**Figure 1:**
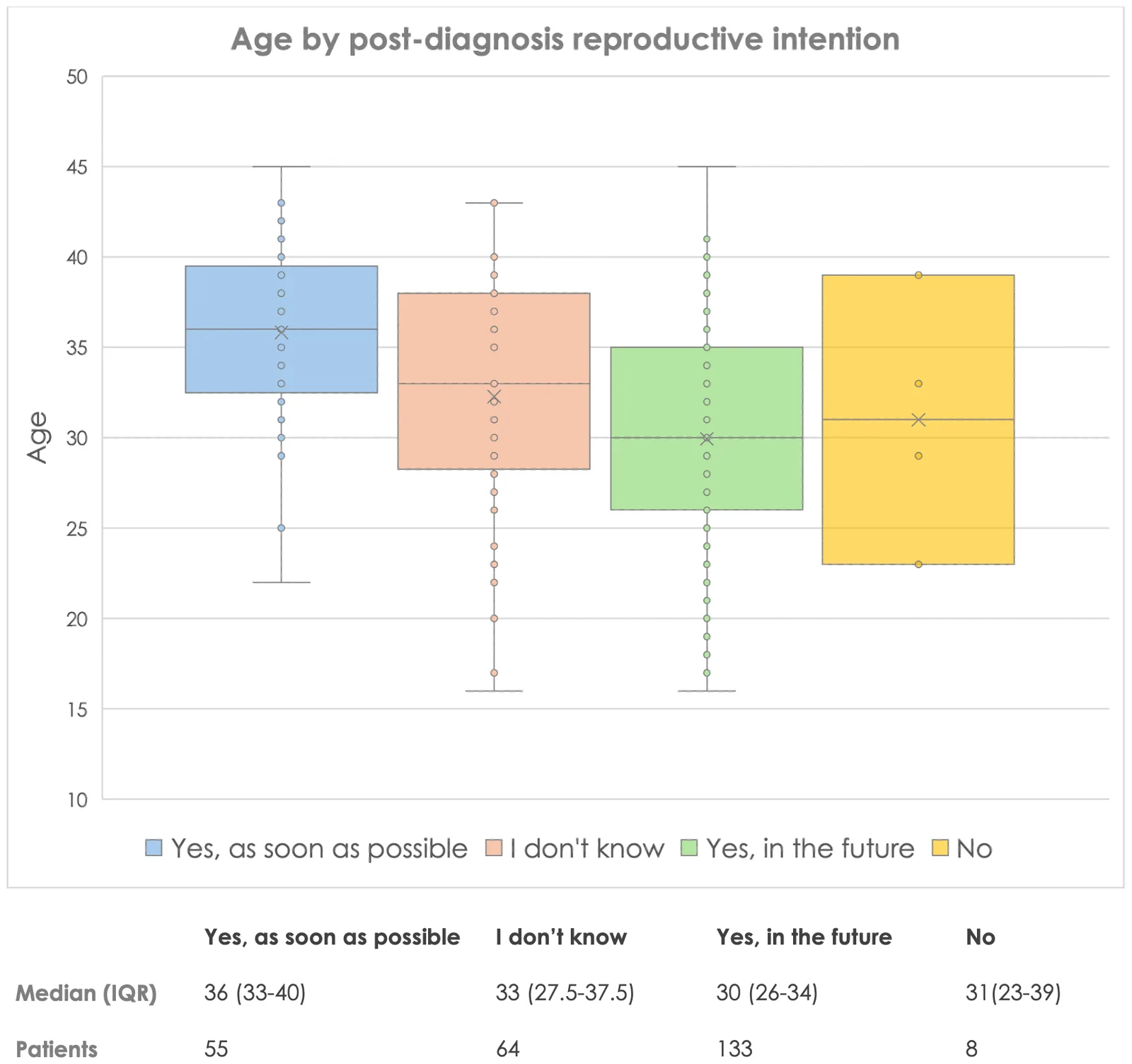
Age distribution according to post-diagnosis reproductive intention. Boxplots showing the distribution of age across the four categories of post-diagnosis reproductive intention (“Yes, as soon as possible”, “I don’t know”, “Yes, in the future”, and “No”). For each group, the box represents the interquartile range, the horizontal line indicates the median, whiskers indicate the range, and individual dots represent single observations. Mean age (standard deviation) and the number of patients in each group are reported below the plot.

Post-diagnosis reproductive intention was also significantly associated with relationship status (Fisher’s exact p < 0.001). Among women who were in a relationship, the proportion expressing a positive reproductive intention was higher (76.6%), with 28.3% wishing to conceive as soon as possible and 48.3% wishing to conceive in the future. By contrast, among women who were not in a relationship, the majority either wished to conceive in the future (62.0%) or were uncertain (32.0%) (**Figure 2**).

**Figure 2:**
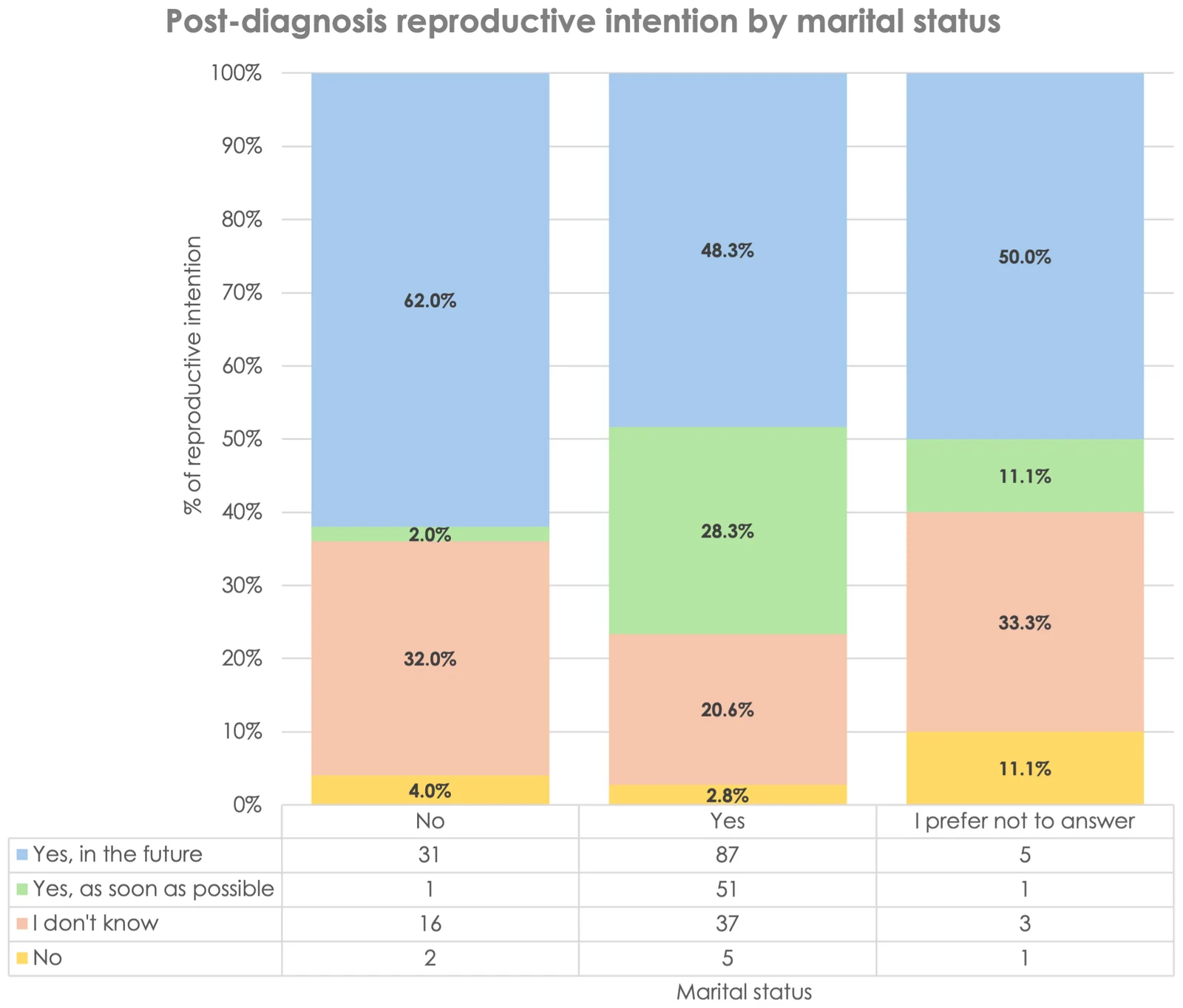
Post-diagnosis reproductive intention according to relationship status. Stacked bar chart showing the distribution of post-diagnosis reproductive intention by relationship status. Percentages are displayed within each bar, and absolute frequencies are reported in the table below the chart.

A significant association was also observed between post-diagnosis reproductive intention and parity (Fisher’s exact p=0.006). Most women without children expressed a positive reproductive intention after diagnosis, with 43/208 (20.7%) wishing to conceive as soon as possible and 111/208 (53.4%) in the future, whereas uncertainty and lack of reproductive intention were more frequent among women who had already had children (**Figure 3**).

**Figure 3:**
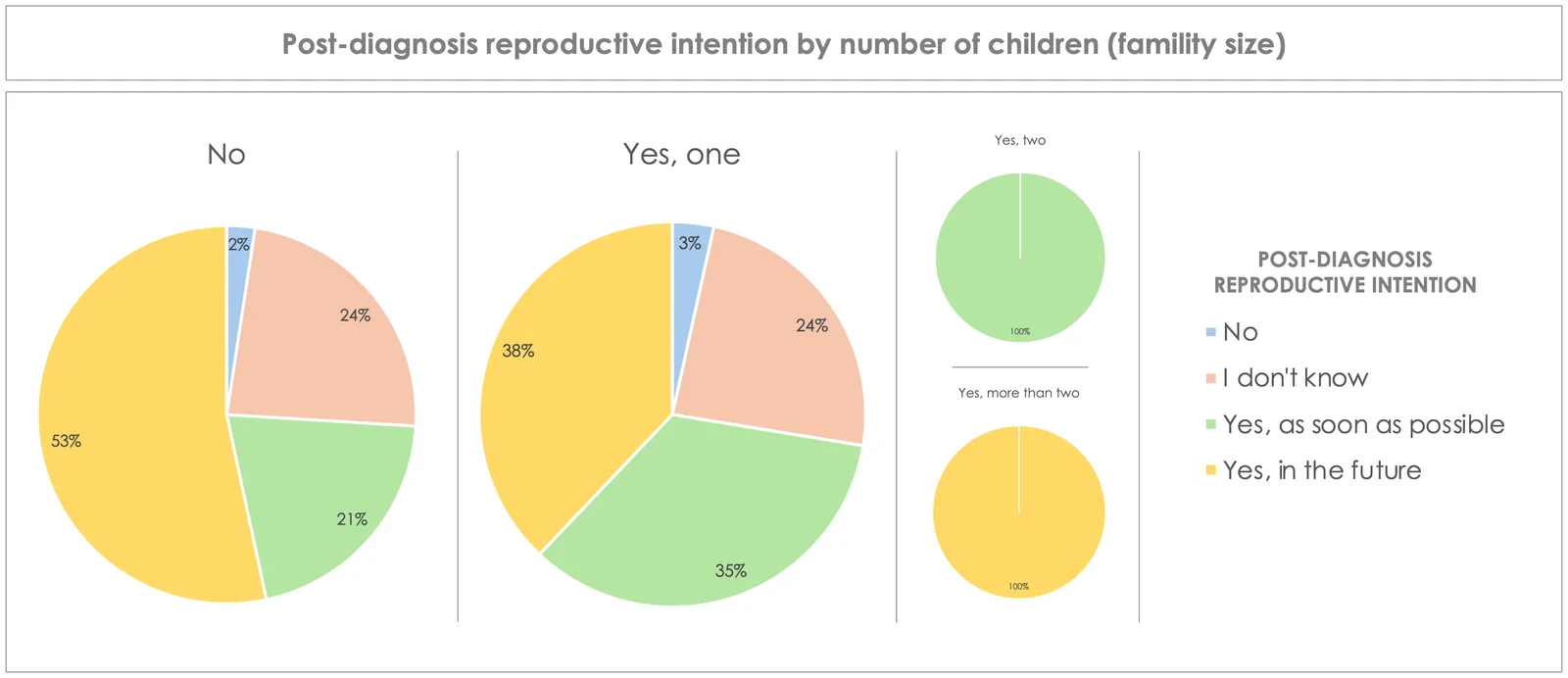
Post-diagnosis reproductive intention according to family size. Pie charts showing the distribution of post-diagnosis reproductive intention according to parity category (no children, one child, two children, and more than two children). Percentages are reported for each response category within each subgroup.

Finally, the distribution of post-diagnosis reproductive intention varied across tumor types. Future reproductive intention predominated among women with hematological malignancies (25/35, 71.4%) and borderline ovarian tumors (22/34, 64.7%), and was also the most frequent response in breast (41/86, 47.7%), cervical (6/12, 50.0%), and invasive ovarian cancers (18/37, 48.7%). On the other hand, immediate reproductive intention was the most frequent response in endometrial cancer (7/12, 58.3%), whereas uncertainty was particularly frequent in the unspecified cancer group (5/8, 62.5%). Given the very small numbers in several tumor-specific subgroups, these findings should be interpreted descriptively (**Supplementary Material**, **Supplementary Figure S1**).

### 3.6 Multivariable logistic regression

In multivariable logistic regression analysis, post-diagnosis reproductive intention was dichotomized into a definite positive reproductive intention (“yes, as soon as possible” or “yes, in the future”) versus no or uncertain reproductive intention (“no” or “I do not know”). The model, based on 188 complete cases, was statistically significant overall (LR χ²=40.65, p=0.0004), with a pseudo-R² of 0.1923, indicating limited model performance. Pre-diagnosis reproductive intention showed the strongest independent association: women with a pre-diagnosis desire for pregnancy had higher odds of definite positive post-diagnosis reproductive intention after diagnosis (OR 11.64, 95% CI 4.45–30.48; p<0.001).

Compared with the ≤21-year reference group, women aged 34–39 years had lower odds, but this isolated category estimate was imprecise (OR 0.07, 95% CI 0.01–0.79; p=0.032) and should be interpreted cautiously; no consistent association was seen across other age categories.

No significant independent associations were found for employment status, relationship status, parity, partner involvement in fertility preservation decisions, uncertainty/perplexity on fertility preservation, or perceived impact of chemotherapy on fertility preservation (**Table 2**).

**Table 2:** Multivariable logistic regression analysis of factors associated with definite positive post-diagnosis reproductive intention.

Variable	Category	aOR (95% CI)	P-value
Age category	≤21 years	Reference	—
	22–27 years	0.12 (0.01–1.50)	0.101
	28–33 years	0.13 (0.02–1.61)	0.115
	34–39 years	0.07 (0.01–0.79)	0.032
	>39 years	0.16 (0.01–2.93)	0.219
Employment status	No	Reference	—
	Yes	1.05 (0.71–1.57)	0.803
Relationship status	No relationship	Reference	—
	In a relationship	1.62 (0.39–6.63)	0.504
Having children	No	Reference	—
	Yes	1.01 (0.36–2.91)	0.972
Partner involvement in fertility-preservation decisions	Not at all	Reference	—
	Slightly	3.87 (0.59–25.21)	0.157
	Moderately	1.49 (0.33–6.79)	0.607
	Very	1.48 (0.34–6.53)	0.605
	Extremely	1.12 (0.28–4.39)	0.873
Pre-diagnosis reproductive intention	No	Reference	—
	Yes	11.64 (4.45–30.48)	<0.001
Uncertainty/perplexity on fertility preservation	No	Reference	—
	Yes	0.73 (0.33–1.64)	0.452
Perceived influence of chemotherapy on fertility preservation	No	Reference	—
	I don’t know	0.97 (0.34–2.78)	0.957
	Yes	0.96 (0.39–2.36)	0.921

Model summary. Number of observations = 188; LR χ²(15) = 40.65; p = 0.0004; pseudo-R² = 0.1923; log likelihood = -85.39416.

aOR, adjusted odds ratio; CI, confidence interval; LR, likelihood ratio.

## 4 Discussion

### 4.1 Main findings

This cross-sectional survey largely confirms patterns reported in oncofertility literature and contextualizes them within an Italian tertiary referral setting. In this cohort, awareness that cancer treatment may impair fertility was high and FP information highly valued; however, general awareness did not consistently translate into knowledge of available options. Almost one in four women reported no knowledge of any FP strategy, and awareness was concentrated on oocyte cryopreservation, with much lower awareness of ovarian tissue or embryo cryopreservation. Thus, the study mainly confirms and contextualizes this awareness–knowledge gap within a selected Italian referral cohort; nearly half received information from non-medical sources, including relatives and media.

Attitudes towards FP showed strong interest but substantial uncertainty. Before diagnosis, most women had planned future childbearing; after diagnosis, positive intention remained common, but responses shifted toward greater uncertainty, particularly among women without prior intentions. This descriptive shift is clinically informative and should not be interpreted as a definitive change or a specific psychological response. Concern about transmitting cancer to offspring contrasts with available evidence (32). concerns regarding treatment delay, safety, effectiveness, costs and possible interference with oncological treatment. These concerns persist despite evidence and guidelines indicating that fertility preservation can usually be performed without clinically relevant delays, including random-start ovarian stimulation protocols, while ovarian tissue cryopreservation remains an option when treatment cannot be postponed (9, 20, 33, 34). In Italy, fertility preservation is covered by the National Health Service, although patient awareness of this provision may be limited (35).

Overall, counselling should move beyond presenting FP options and elicit prior intentions. It should address the main concerns identified here: timing, safety, effectiveness and costs. Uncertain patients may benefit from reassessment as clinical information becomes clearer, when treatment timelines permit (36, 37).

### 4.2 Strengths and limitations

The present study stands out for several methodological and clinical reasons. First, the questionnaire was developed through a rigorous two-round Delphi process involving multidisciplinary experts, ensuring high content validity (CVI >79%). Second, the high response rate (91.5%) provides a robust snapshot of women referred for fertility preservation counselling. Third, by administering the survey before the specialist visit, we captured the “raw” fears and needs of the patients, providing a unique window into the psychological state that oncologists and gynecologists must address during the first encounter.

Some limitations should be acknowledged, including cross-sectional design and self-report bias. Recall and social desirability bias are also possible. All participants had been referred for FP consultation at a single tertiary center, making referral an important source of selection bias. Referred women may differ from non-referred patients in features, concerns or access. The denominator of eligible women in referring oncology services was unknown, preventing estimation of referral proportion or comparison of referred and non-referred patients. Findings therefore apply mainly to referred patients in similar tertiary settings. Delphi content validity was established, but the 50-item survey may impose burden. Regression used 188/240 complete cases; Missing-data bias is therefore possible.

### 4.3 Current literature

Our findings broadly confirm prior surveys while reflecting the selected referral context. In a multicenter cross-sectional study of women with breast cancer, Ko et al. reported that only 44.1% had heard of fertility preservation and that fertility concerns influenced treatment decisions in 46.1% of cases, with awareness associated with younger age and higher educational level (23). Similarly, a Brazilian population-based survey showed limited baseline knowledge, with only 34.0% aware that cancer treatment could impair fertility and 22.0% aware of fertility preservation options (24). By contrast, the high awareness observed in our cohort (89.6%) likely reflects referral to a dedicated fertility preservation consultation, as well as increasing oncofertility visibility over time.

Previous studies also suggest that information provision does not necessarily ensure adequate understanding or recall. Banerjee and Tsiapali reported documented fertility discussions in only 55% of young breast cancer patients, and only 52% of counselled patients recalled the conversation (25). More recently, Sbaity et al. found that although 87.8% of women with breast cancer or lymphoma had been informed about fertility risks, only 45.4% desired fertility preservation, with uptake influenced by age, parity and educational level (26). Overall, these data support the interpretation that fertility preservation is a highly valued but complex decision, shaped not only by access to information but also by timing, emotional distress, parity, education and counselling quality.

### 4.4 Future perspectives and clinical implications

Future efforts should move beyond simply increasing awareness and focus on implementing structured, timely and equitable oncofertility pathways. Current guidelines recommend discussing infertility risk as early as possible and ensuring prompt referral to fertility specialists before gonadotoxic treatment whenever feasible (9). However, real-world implementation remains inconsistent. Dedicated oncofertility programs have been shown to improve recall of fertility discussions and referral rates without delaying systemic treatment (28), while qualitative evidence indicates that healthcare professionals may still feel uncertain about their role, lack confidence in their knowledge, or be influenced by assumptions related to age, parity or ethnicity when addressing fertility preservation (27).

A clinically relevant descriptive finding is the shift toward uncertainty after diagnosis. Rather than indicating a definite preference change, it suggests that goals may be less settled immediately after diagnosis and may warrant reassessment when clinically feasible. Pre-diagnosis intention showed the strongest model association (OR 11.64, 95% CI 4.45–30.48), but limited model performance (pseudo-R²=0.19) and wide confidence intervals warrant cautious interpretation. The isolated lower odds in women aged 34–39 years may reflect age-related feasibility concerns rather than “pragmatic resignation”; motivation was not directly assessed, so a psychological interpretation is not supported.

Clinical pathways should translate these findings into concrete steps. At the first oncology or FP discussion, clinicians should elicit and document pre-diagnosis reproductive intentions and provide concise, option-specific information rather than relying on general awareness. Counselling should explicitly address treatment timing, procedural safety, effectiveness and costs or coverage, which were common concerns in this cohort. Patients expressing uncertainty should be offered reassessment as clinical information becomes clearer, when timelines permit. Prompt coordination between oncology and fertility specialists should minimize avoidable referral delay, while psychological support should be offered according to expressed need. In women with gynecological cancers, recommendations should account for tumor-specific feasibility and oncological safety (30). Implementation studies should assess timely referral, decisional quality and appropriate FP uptake; and longer-term reproductive and psychosocial outcomes.

### 4.5 Conclusions

These findings confirm and contextualize prior evidence within an Italian tertiary referral cohort. The single-center setting and shift toward uncertainty should frame interpretation. Pre-diagnosis intention was strongly associated with post-diagnosis goals, supporting early elicitation of reproductive goals, option-specific counselling on timing, safety and costs, and reassessment of uncertain preferences. As the referral denominator was unknown, findings apply mainly to similar tertiary settings and should be validated in broader populations.

## Data Availability

The original contributions presented in the study are included in the article/**Supplementary Material**. Further inquiries can be directed to the corresponding author.
