# Supplementary material for: Fertility preservation counselling in women with cancer: knowledge, preferences, and unmet needs from a cross-sectional survey
Source: Front Endocrinol (Lausanne). 2026 Oct 6;17:1926475. doi: 10.3389/fendo.2026.1926475 (PMC13684863; doi:10.3389/fendo.2026.1926475)
Supplement: Supplementary file 1 [file SupplementaryFile1.docx]

Supplementary Material

# Table S1. Survey questions

| **Item** | **Question** | **Response options / field type** |
| --- | --- | --- |
| **SOCIO-DEMOGRAPHIC INFORMATION** | | |
| 1.1 | What is your age? | Open field |
| 1.2 | Nationality: | Open field |
| 1.3 | If you live in Italy, in which Italian region do you currently reside? | Open field |
| 1.4 | Are you currently in a relationship? | • No, I am single  • Yes, I am casually dating a partner  • Yes, I am in a stable relationship  • Yes, I am cohabiting / living with a partner  • Yes, I am married  • No, I am separated/divorced  • I prefer not to answer |
| 1.5 | Level of education | • Primary school diploma  • Middle school diploma  • High school diploma  • University degree  • Postgraduate education (e.g. Master’s degree, PhD, etc.) |
| 1.6 | Do you currently have paid employment? | • Yes, full-time  • Yes, part-time  • No  • I prefer not to answer |
| **CLINICAL HISTORY** | | |
| 2.1 | What type of cancer have you been diagnosed with? | Open field |
| 2.2 | Stage of disease: | • I  • II  • III  • IV  • Unknown  • I do not know |
| 2.3 | Age at diagnosis: | Open field |
| 2.4 | How many weeks elapsed between your cancer diagnosis and your first fertility preservation consultation? | Open field |
| 2.5 | Are you aware of any blood relatives who have been diagnosed with cancer? | • Yes  • No  • I do not know |
| 2.6 | Are you currently receiving, or have you received, hormonal treatment after your cancer diagnosis? | • Yes  • No  • I do not know / I do not remember |
| 2.7 | If yes, which treatment(s)? | Open field |
| 2.8 | If yes, for how long? | • <2 years  • 2–5 years  • >5 years  • I do not know / I do not remember |
| 2.9 | Do you have any other chronic medical conditions? | • None  • Diabetes  • Kidney disease  • Obesity  • Hypertension  • Cardiovascular disease  • Anaemia  • Coagulation disorders  • Asthma  • Pulmonary disease  • Inflammatory bowel disease  • Coeliac disease  • Epilepsy  • Hypothyroidism/Hyperthyroidism  • Autoimmune disease  • Other. Please specify: __________  • I do not know |
| 2.10 | What was your weight in kg before your cancer diagnosis? | Open field |
| 2.11 | What is your current weight in kg? | Open field |
| 2.12 | What is your height in cm? | Open field |
| **FERTILITY AND COUNSELLING** | | |
| 3.1 | In your opinion, could chemotherapy/radiotherapy reduce your fertility? | • Yes  • No |
| 3.2 | In your opinion, how can chemotherapy/radiotherapy affect fertility? | • Chemotherapy/radiotherapy always reduces fertility  • Chemotherapy/radiotherapy does not always reduce fertility  • Chemotherapy/radiotherapy never reduces fertility  • I do not know |
| 3.3 | Have you ever heard about fertility preservation? | • Yes  • No |
| 3.4 | If yes, when did you first hear about fertility preservation? | • Before the cancer diagnosis  • After the cancer diagnosis |
| 3.5 | If yes, from whom did you hear about fertility preservation? | Multiple choice: Specialist at the time of cancer diagnosis; Oncologist after the diagnosis; General practitioner / family doctor; Friends / relatives; Media (web, newspapers, TV, etc.); Other specialist |
| 3.6 | How important was it for you to receive information about fertility preservation? | Likert scale: 1 = Not at all; 2 = Slightly; 3 = Moderately; 4 = Very; 5 = Extremely |
| 3.7 | Which of the following fertility preservation options are you aware of? | • Embryo cryopreservation  • Oocyte cryopreservation  • Ovarian tissue cryopreservation  • Gonadotropin-releasing hormone agonists  • None |
| 3.8 | In your opinion, which healthcare professional should provide information about fertility preservation to patients? | • Oncologist  • Surgeon  • General practitioner / family doctor  • Physician/specialist supported by a psychologist  • Gynaecologist and/or reproductive specialist  • Other. Please specify: __________ |
| 3.9 | In your opinion, which women diagnosed with cancer should receive information about fertility preservation? | • Women without children who wish to have children  • Women who already have children and wish to have more children  • Women of reproductive age  • No women  • All women |
| 3.10 | Do you have any concerns about fertility preservation? | • Yes  • No |
| 3.11 | If yes, what kind of concerns? | • High costs  • Ethical issues  • Effectiveness  • Safety  • Delay in the start of cancer treatment  • Other. Please specify: __________ |
| 3.12 | In your opinion, why is it important to receive information about fertility preservation? | Multiple choice: Because it leads to a better understanding of fertility preservation issues; Because it improves future fertility prospects; Because it improves the relationship with one’s partner; Because it leads to more informed decisions; I do not consider it a priority at the moment, given my cancer diagnosis; Other. Please specify: __________ |
| 3.13 | In your opinion, can fertility preservation, which must be completed before the start of chemotherapy/radiotherapy, influence cancer treatment? | • Yes  • No  • I do not know |
| 3.14 | If yes, in what way? | • Because it delays the start of cancer treatment  • Because of possible complications related to ovarian tissue or oocyte retrieval procedures  • Because it helps patients face the treatment pathway more calmly  • I do not know |
| 3.15 | Regarding the decision to preserve fertility, would you prefer that: | • The doctor makes the decision without consulting you  • The doctor makes the final decision after considering your opinion  • You and the doctor decide together  • You make the decision yourself after considering your doctor’s opinion  • You make the decision yourself without consulting your doctor |
| 3.16 | Do you know anyone who has undergone fertility preservation? | • Yes  • No |
| 3.17 | If no, would you like to know about other women’s experiences before making your own decision? | • Yes  • No |
| 3.18 | How involved is your partner in decisions regarding fertility preservation treatment? | Likert scale: 1 = Not at all; 2 = Slightly; 3 = Moderately; 4 = Very; 5 = Extremely |
| 3.19 | Would you currently be interested in starting a fertility preservation procedure? | • Yes  • No  • I do not know |
| 3.20 | Do you have any suggestions on how information about fertility preservation should be provided? | Open field |
| **REPRODUCTIVE ATTITUDES** | | |
| 4.1 | Have you ever been pregnant? | • Yes  • No |
| 4.2 | Do you have children? | • Yes, one  • Yes, two  • Yes, more than two  • No |
| 4.3 | Before your cancer diagnosis, were you planning to have children / more children? | • Yes  • No |
| 4.4 | After your diagnosis, are you planning to have children / more children? | • Yes, as soon as possible  • Yes, in the future  • No  • I do not know |
| 4.5 | If, after your cancer diagnosis, you do not wish to have children in the future, why? | • Because I would not be able to take care of them if the cancer recurred  • Because fertility preservation may affect my recovery  • Because pregnancy might increase the risk of recurrence  • Because I think cancer treatment could negatively affect the health of my future children  • Because I am afraid of passing cancer on to my children because of genetics or previous chemotherapy exposure |
| 4.6 | Have you or your partner ever received a diagnosis of couple infertility? | • Yes  • No  • I do not have a partner |
| 4.7 | After your cancer diagnosis, have you ever considered foster care or adoption? | • Yes  • No |
| **PERSONAL IMPLICATIONS** | | |
| 5.1 | What are your thoughts mainly focused on at this moment? | Multiple choice: The start of cancer treatment; Symptoms; Fear of not being able to have children; The need for clearer information; Fertility preservation options; Feeling sorry for the distress I am causing my partner and loved ones; The feeling of being unable to cope with all of this; Positive aspects related to my resilience and ability to cope |
| 5.2 | After your cancer diagnosis, what could help you feel better? | Multiple choice: Psychological support; Support from friends/family; Joining groups of people who share the same problem; The possibility of undergoing fertility preservation treatment; Other. Please specify: __________; Nothing, I feel fine |
| 5.3 | Do you consider psychological support important at this time? | • Yes  • No |
| 5.4 | With whom would you prefer to attend the fertility preservation counselling visit? | • Alone  • With my family members  • With my partner  • With a significant non-family person (e.g. friend, colleague)  • I do not know |
| 5.5 | Has your cancer diagnosis affected your relationship with your partner? | • Yes, positively  • Yes, negatively  • No  • I am not currently in a relationship |

**Table S2.** Baseline socio-demographic and clinical characteristics, fertility preservation knowledge, preferences, and unmet needs of the study population

| **Consent** | 240/240 (100) |
| --- | --- |
| **Age in years** | Median 32 (IQR 27-37) |
| **Nationality**  Italian  Others | 206/240 (85.8)  34/240 (14.2) |
| **Relationship**  No, I am separated/divorced  No, I am single  Yes, I am casually dating a partner  Yes, I am in a stable relationship  Yes, I am cohabiting / living with a partner  Yes, I am married  I prefer not to answer | 1/240 (0.4)  49/240 (20.4)  10/240 (4.2)  61/240 (25.4)  69/240 (28.7)  40/240 (16.7)  10/240 (4.2) |
| **Level of education**  Middle school diploma  High school diploma  University degree  Postgraduate education (e.g. Master’s Degree, PhD, etc.) | 15/240 (6.2)  76/240 (31.7)  94/240 (39.2)  55/240 (22.9) |
| **Employment status**  No  Yes, full-time  Yes, part-time  I prefer not to answer | 68/240 (28.3)  131/240 (54.6)  34/240 (14.2)  7/240 (2.9) |
| **Disease**  Borderline ovarian cancer  Invasive ovarian cancer  Endometrial cancer  Uterine cancer  Cervical cancer  Vulvar cancer  Breast cancer  Hematological malignancy  Colorectal cancer  Lung cancer  Brain tumor  Skin cancer  Cancer of uncertain origin  Unspecified cancer | 34/240 (14.2)  37/240 (15.4)  12/240 (5.0)  2/240 (0.8)  12/240 (5.0)  1/240 (0.4)  86/240 (35.9)  35/240 (14.6)  6/240 (2.5)  1/240 (0.4)  3/240 (1.3)  2/240 (0.8)  1/240 (0.4)  8/240 (3.3) |
| **Stage of disease**  I  II  III  IV  Unknown | 47/240 (19.6)  33/240 (13.7)  28/240 (11.7)  7/240 (2.9)  125/240 (52.1) |
| **Age at diagnosis in years*** availbale in 239/240 | Median 32 (IQR 26-37) |
| **Time in weeks from cancer diagnosis to first fertility preservation consultation*** availbale in 227/240 | Median 4 (IQR 2-14) |
| **Family history of cancer**  Yes  No  Unknown | 142/240 (59.2)  83/240 (34.6)  15/240 (6.2) |
| **Ormonal therapy**  Yes  No  Unknown | 37/240 (15.4)  191/240 (79.6)  12/240 (5.0) |
| **Specific ormonal therapy**  *GnRH agonists*  Triptorelin (Decapeptyl)  Leuprorelin (Enantone)  *GnRH agonists and aromatase inhibitors*  Triptorelin and Letrozole  Triptorelin and Exemestane  Leuprorelin and Exemestane  *GnRH agonists and SERM*  Triptorelin and Tamoxifen  *Progestins / progestational agents*  Progesterone  Medroxyprogesterone acetate (Farlutal)  Megestrol acetate (Megace)  *SERM*  Tamoxifen  *LNG-IUD and Progestins / progestational agents*  LNG-IUD and Megestrol acetate  *Combined hormonal therapy*  Estroprogestin pill  Estradiol/dydrogesterone (Femoston)  *Not specified* | 4/37 (10.8)  7/37 (18.9)  3/37 (8.1)  3/37 (8.1)  2/37 (5.4)  1/37 (2.7)  4/37 (10.8)  1/37 (2.7)  1/37 (2.7)  1/37 (2.7)  1/37 (2.7)  2/37 (5.4)  2/37 (5.4)  5/37 (13.6) |
| **Duration of hormonal therapy**  < 2 years  2-5 years  > 5 years  Unknown | 21/37 (56.8)  8/37 (21.6)  5/37 (13.5)  3/37 (8.1) |
| **Chronic disease** *available in 226/240  Alopecia areata  Anemia  Arthrosis  Asthma  Autoimmune disease  Celiac disease  Diabetes  Endometriosis  Epilepsy  Favism  Fibromyalgia  Gastritis  Hypothyroidism/Hyperthyroidism  Insulin resistance  Kidney disease  Obesity  Thrombocytopenia  Psoriasis  Retinitis pigmentosa  Multiple sclerosis  Gilbert syndrome  Thrombophilia  Vulvodynia  None | 1/226 (0.4)  5/226 (2.2)  1/226 (0.4)  3/226 (1.3)  4/226 (1.8)  1/226 (0.4)  1/226 (0.4)  2/226 (0.9)  1/226 (0.4)  1/226 (0.4)  3/226 (1.3)  2/226 (0.9)  21/226 (9.3)  2/226 (0.9)  1/226 (0.4)  2/226 (0.9)  1/226 (0.4)  1/226 (0.4)  1/226 (0.4)  1/226 (0.4)  1/226 (0.4)  1/226 (0.4)  1/226 (0.4)  168/226 (74.4) |
| **Height in centimeters** | Mean 164.6 (±6.4) |
| **Pre-diagnosis weight in kg** *available in 236/240 | Median 60 (IQR 53-66) |
| **Current weight in kg** *available in 238/240 | Median 60 (IQR 53-65) |
| **BMI category** *available in 238/240  Underweight (<18.5 kg/m^2^)  Healthy weight (18.5-24.9 kg/m^2^)  Overweight (25.0-29.9 kg/m^2^)  Obesity ( ≥ 30.0 kg/m^2^) | 28/238 (11.8)  157/238 (66.0)  36/238 (15.1)  17/238 (7.1) |
| **In your opinion, could chemotherapy/radiotherapy reduce your fertility?**  Yes  No | 226/240 (94.2)  14/240 (5.8) |
| **In your opinion, how can chemotherapy/radiotherapy affect fertility?**  Chemotherapy/radiotherapy never reduces fertility  Chemotherapy/radiotherapy does not always reduce fertility  Chemotherapy/radiotherapy always reduces fertility  I don’t know | 1/240 (0.4)  94/240 (39.2)  72/240 (30.0)  73/240 (30.4) |
| **Have you ever heard about fertility preservation?**  Yes  No | 215/240 (89.6)  25/240 (10.4) |
| **If yes, when did you hear about fertility preservation?**  Before cancer diagnosis  After cancer diagnosis | 104/240 (48.4)  111/215 (51.6) |
| **If yes, when did you first hear about fertility preservation?**  Friends/relatives  Media (web, newspapers, TV, etc.)  General practitioner  Specialist at the time of cancer diagnosis  Oncologist after diagnosis  Other specialists | 80/373 (21.4)  79/373 (21.2)  14/373 (3.8)  82/373 (22.0)  88/373 (23.6)  30/373 (8.0) |
| **How important was it for you to receive information about fertility preservation?** *available in 229/240  Not at all  Slightly  Moderately  Very  Extremely | 1/229 (0.4)  6/229 (2.6)  37/229 (16.2)  60/229 (26.2)  125/229 (54.6) |
| **Which of the following fertility preservation options are you aware of?**  GnRH agonists  Oocyte cryopreservation  Ovarian tissue cryopreservation  Embryo cryopreservation  None | 2/240 (0.8)  154/240 (64.2)  17/240 (7.1)  9/240 (3.7)  58/240 (24.2) |
| **In your opinion, which healthcare professional should provide patients with information about fertility preservation?**  Gynecologist and/or reproductive specialist  General practitioner  Physician/specialist supported by a psychologist  Oncologist  All of the above | 132/240 (55.0)  5/240 (2.1)  39/240 (16.2)  61/240 (25.4)  3/240 (1.3) |
| **In your opinion, which women diagnosed with cancer should receive information about fertility preservation?**  Women who do not have children and wish to have children  Women of reproductive age  All women | 24/240 (10.0)  77/240 (32.1)  139/240 (57.9) |
| **Do you have any concerns about fertility preservation?**  Yes  No | 133/240 (55.4)  107/240 (44.6) |
| **If yes, what kind of concerns?**  Costs  Effectiveness  Costs and effectiveness  Safety  Pain  Delay in the initiation of cancer treatment  Lack of knowledge  Ethical issues  Post-treatment consequences  Unspecified | 26/133 (19.5)  25/133 (18.8)  1/133 (0.8)  30/133 (22.6)  1/133 (0.8)  39/133 (29.3)  1/133 (0.8)  6/133 (4.5)  1/133 (0.8)  3/133 (2.3) |
| **In your opinion, why is it important to receive information about fertility preservation? (more than one answer may be selected)**  Because it leads to a better understanding of fertility preservation issues  Because it helps patients make more informed decisions  Because it improves future fertility prospects  Because it improves the relationship with one’s partner  I do not consider it a priority at the moment given my cancer diagnosis | 171/486 (35.2)  161/486 (33.1)  118/486 (24.3)  32/486 (6.6)  4/486 (0.8) |
| **In your opinion, can fertility preservation, which must be completed before the start of chemotherapy/radiotherapy, affect cancer treatment?**  Yes  No  I don’t know | 83/240 (34.6)  53/240 (22.1)  104/240 (43.3) |
| **If yes, in what way?**  Unspecified  Because it helps patients face the treatment pathway with greater peace of mind  Because of the possible complications of the procedures used for ovarian tissue and oocyte retrieval  Because it delays the start of cancer treatment | 2/83 (2.4)  7/83 (8.4)  36/83 (43.4)  38/83 (45.8) |
| **Regarding the decision to preserve fertility, would you prefer that:**  You and your doctor make the decision together  You make the decision yourself, after considering your doctor’s opinion  The doctor makes the final decision, after considering your opinion  You make the decision yourself, without consulting your doctor | 112/240 (46.7)  119/240 (49.6)  1/240 (0.4)  8/240 (3.3) |
| **Do you know anyone who has undergone fertility preservation?**  Yes  No | 55/240 (22.9)  185/240 (77.1) |
| **If not, would you like to know about other women’s experiences before making your decision?** *available for 173/185  Yes  No | 128/173 (74.0)  45/173 (26.0) |
| **How involved is your partner in decisions regarding fertility preservation treatment? (only for women who have a partner)**  Not at all  Slightly  Moderately  Very  Extremely | 25/190 (13.2)  20/190 (10.5)  36/190 (18.9)  41/190 (21.6)  68/190 (35.8) |
| **Would you be interested in starting a fertility preservation procedure at this time?**  Yes  No  I don’t know | 167/240 (69.6)  11/240 (4.6)  62/240 (25.8) |
| **Do you have any suggestions on how information about fertility preservation should be provided? *available for 237/240**  No suggestions  Consultation with a specialist physician  Information dissemination / awareness campaigns  Clarity  At the time of diagnosis  Empathy  General practitioners | 122/237 (51.5)  45/237 (19.0)  28/237 (11.8)  20/237 (8.4)  17/237 (7.2)  4/237 (1.7)  1/237 (0.4) |
| **Have you ever been pregnant?**  Yes  No | 46/240 (19.2)  194/240 (80.8) |
| **Do you have children?**  No  Yes, one  Yes, two  Yes, more than two | 208/240 (86.7)  29/240 (12.1)  2/240 (0.8)  1/240 (0.4) |
| **Before the cancer diagnosis, were you planning to have children/more children?**  Yes  No | 177/240 (73.7)  63/240 (26.3) |
| **After the diagnosis, are you planning to have children/more children?**  No  I don’t know  Yes, as soon as possible  Yes, in the future | 8/240 (3.3)  56/240 (23.3)  53/240 (22.1)  123/240 (51.3) |
| **If, after the cancer diagnosis, you do not wish to have children in the future, why?** *available 44/64  Because fertility preservation may affect my recovery  Because I am afraid of passing the cancer on to my children due to genetics or previous exposure to chemotherapy  Because I might not be able to take care of them if the cancer were to recur  Because pregnancy might increase the risk of recurrence  Because I think cancer treatment could negatively affect the health of my future children | 4/44 (9.1)  13/44 (29.6)  9/44 (20.4)  11/44 (25.0)  7/44 (15.9) |
| **Have you and your partner ever received a diagnosis of couple infertility?**  Yes  No  I don’t have a partner | 10/240 (4.2)  175/240 (72.9)  55/240 (22.9) |
| **After the cancer diagnosis, have you ever considered foster care or adoption?**  Yes  No | 83/240 (34.6)  157/240 (65.4) |
| **What is your mind focused on at this moment?**  Start of cancer treatment  Fear of not being able to have children  Fertility preservation options  Positive aspects related to my resilience and ability to cope  Need for clearer information  Feeling sorry for the distress I am causing my partner and loved ones  Feeling of being unable to cope with all of this  Symptoms | 107/602 (17.7)  103/602 (17.1)  94/602 (15.6)  91/602 (15.1)  84/602 (14.0)  51/602 (8.5)  44/602 (7.3)  28/602 (4.7) |
| **After the cancer diagnosis, what could help you feel better?**  Support from friends/family  Psychological support  Possibility of undergoing fertility preservation treatment)  Joining support groups with people sharing the same condition  Nothing, I already feel well  Resuming physical activity  Alternatives to avoid chemotherapy | 149/473 (31.5)  135/473 (28.5)  109/473 (23.0)  42/473 (8.9)  35/473 (7.4)  1/473 (0.2)  2/473 (0.4) |
| **Do you consider psychological support important at this time?**  Yes  No | 191/240 (79.6)  49/240 (20.4) |
| **With whom would you prefer to be during the fertility preservation consultation?**  Together with my partner  Together with my family members  Alone  Together with a trusted person outside the family (e.g. friend, colleague)  I don’t know | 120/313 (38.3)  89/313 (28.4)  68/313 (21.7)  22/313 (7.0)  14/313 (4.5) |
| **Has the cancer diagnosis affected your relationship with your partner?**  No  Yes, positively  Yes, negatively  I don’t have a relationship | 120/240 (50.0)  44/240 (18.4)  26/240 (10.8)  50/240 (20.8) |

*****IQR = Interquartile Range

**Figure S1. Post-diagnosis reproductive intention according to tumour type.** Upper panel: horizontal bar chart showing the absolute number of patients in each post-diagnosis reproductive intention category by tumour type. Lower panel: stacked bar chart showing the corresponding percentage distribution within each tumour type.
